# How well can morphology assess cell death modality? A proteomics study

**DOI:** 10.1038/cddiscovery.2016.68

**Published:** 2016-10-03

**Authors:** Alexey L Chernobrovkin, Roman A Zubarev

**Affiliations:** 1 Division of Physiological Chemistry I, Department of Medical Biochemistry and Biophysics, Karolinska Institutet, Scheelesväg 2, SE-17 177 Stockholm, Sweden

## Abstract

While the focus of attempts to classify cell death programs has finally shifted in 2010s from microscopy-based morphological characteristics to biochemical assays, more recent discoveries have put the underlying assumptions of many such assays under severe stress, mostly because of the limited specificity of the assays. On the other hand, proteomics can quantitatively measure the abundances of thousands of proteins in a single experiment. Thus proteomics could develop a modern alternative to both semiquantitative morphology assessment as well as single-molecule biochemical assays. Here we tested this hypothesis by analyzing the proteomes of cells dying after been treated with various chemical agents. The most striking finding is that, for a multivariate model based on the proteome changes in three cells lines, the regulation patterns of the 200–500 most abundant proteins typically attributed to household type more accurately reflect that of the proteins directly interacting with the drug than any other protein subset grouped by common function or biological process, including cell death. This is in broad agreement with the 'rigid cell death mechanics' model where drug action mechanism and morphological changes caused by it are bijectively linked. This finding, if confirmed, will open way for a broad use of proteomics in death modality assessment.

## Introduction

Since the first descriptions of programmed cell death mechanisms in 1960s^1–7^ and until 2000s, most attempts to classify cell death programs were based on morphological characteristics. In 1973, Schweichel and Merker proposed a classification of several cell death modalities, including ‘type I cell death’ associated with heterophagy, ‘type II cell death’ associated with autophagy and ‘type III cell death’ not associated with any digestion.^8^ According to modern terminology, these types correspond to apoptosis, autophagy and necrosis, respectively.^9–11^ The Nomenclature Committee on Cell Death (NCCD) has formulated in 2005 and 2009 recommendations for the definition of cell death morphotypes.^12,13^ At the same time, numerous attempts have been ongoing to classify cell death mechanisms according to biochemical assays rather than morphological characteristics.^9,13,14^ In 2012, NCCD has expressed the belief that the time has become appropriate for a novel systematic classification of cell death based on measurable biochemical features.^15^

**Figure 1:**
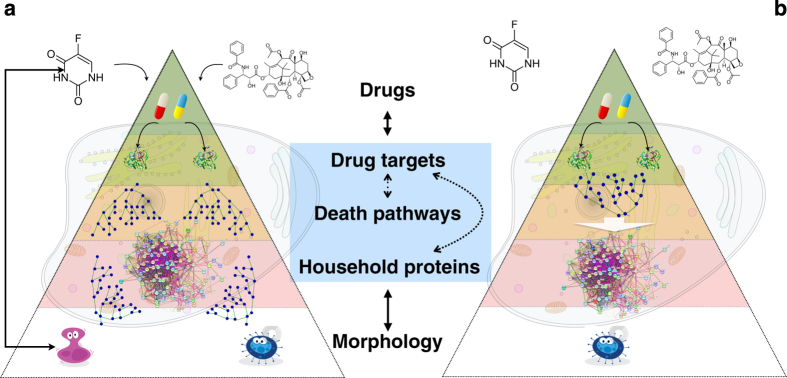
Tight or loose models of cell death regulation. Signal processing analogy can be used – stimulus as an input signal, cellular mechanisms as a black box, top proteome changes as an output signal. Left (**a**): In the tight model, different stimuli cause significantly different states of the dying cell, and such a state can be traced back to the actual cause of death. Right (**b**): In the alternative loose model, different lethal stimuli result in a common, or very similar, state of the dying cell, and based on that state, it is hard to decipher death’s primary cause.

This shift from morphology to biochemistry was intended to signify the relentlessly increasing knowledge of the biochemical features of distinct cell death subroutines. For a long time it has been assumed that morphologically similar states represent the activation of identical or at least similar lethal signaling cascades.^15^ The underlying assumption was the presence of a 'tight' link between the biochemical cascades and morphological changes. But later it has become clear that apparently similar cell death morphological patterns, as assessed by microscopy, can hide a great deal of biochemical heterogeneity. The NCCD has stated that the presence of specific morphological features is not sufficient to establish a causal link between a given process and cellular demise.^15,16^ This statement effectively postulates a 'loose' link between the biochemistry and morphology in cellular death. Figure 1 illustrates the difference between the tight and loose link models. The tight model assumes a bijective link (one-to-one correspondence) between the cause and the effect, that is, between the drug applied and morphological changes in dying cell. Knowing the cell mechanics, this model permits one, at least in principle, to 'reverse-engineer' the death pathway based on the observed morphology. This, in turn, would allow one to identify the upstream area in the protein network that has triggered the corresponding biochemical processes, thus locating the drug target. The loose model makes such a possibility much less probable.

The question whether the cell death mechanics is loose or tight is significant, as the tight mechanics may greatly simplify drug target discovery, for example, by a combination of dynamics proteomics and pathway analysis.^17^ So far, direct comparison of these models has remained challenging, not least because it implies quantitative comparison between the morphology and biochemistry. While biochemistry can be quantitatively assessed by assays employing standards (although single-reaction biochemical readouts are deemed by NCCD to be poor indicators of a precise death modality),^15,16^ morphology assessment has been mostly qualitative and notoriously prone to operator-dependent (mis)interpretations.

A significant challenge for the model comparison is the ever-expanding list of recognized distinct regulated cell death modes. While the 2005 NCCD report listed four main cell death types, 2012 NCCD classification included 13 entries: anoikis, autophagic cell death, caspase-dependent and caspase-independent intrinsic apoptosis, cornification, entosis, extrinsic apoptosis by death receptors and dependence receptors, mitotic catastrophe, necroptosis, netosis, parthanatos and pyroptosis.^15^ These modalities are not mutually exclusive: it was said that a specific cell death-related pathway may progress simultaneously with another cell death mode.^15^ In a 2015 report, NCCD chose not to expand the ever-growing list of cell death modalities and the corresponding recommended assays to characterize them, noting that 'the best biochemical marker of cell death is death itself'.^15^

Such an abrupt conclusion may simply reflect the fact that the plurality of the discovered cell death modalities exceeded the specificity of the methods used for their assessments. A possible solution to this problem could be to increase the analytical specificity of the cell-death probing assay. Here we test whether proteomics can be such an enhanced assay. Indeed, modern proteomics can easily measure the relative abundance of 3000–5000 proteins simultaneously^18–21^ or even of ≥10 000 proteins when larger sample volume and more instrumental time are available.^22–25^ The first few thousands of most abundant proteins constitute more than 90% of proteome mass, thus defining cellular structure and core functions, while the targets of common anticancer drugs are frequently found among the proteins in the low and middle abundance range.^25,26^ Therefore, a conventional 'top proteomics' analysis that takes 1–2 h of instrument time and probes ≤5000 most abundant proteins encompasses both morphology-related proteins as well as drug-interacting molecules.^21,27^

To evaluate the potential of top proteomics in differentiating between similar death modes, and possibly between the loose and tight cell death regulation models, we analyzed the proteomes of three human cancer cell lines after they were treated with five anticancer drugs – 5-fluorouracil (5-FU), methotrexate (MTX), tomudex (TDX), paclitaxel (PCTL) and doxorubicin (DOXO).^28^ These five drugs are widely used worldwide in anticancer therapy and their mechanisms have been studied extensively in last decades. For instance, there are 123 613 papers indexed in Web Of Science (WoS) for 5-FU, 138 613 for MTX, 87 262 for PCTL, 144 278 for DOXO and 915 for TDX. For comparison, for paracetamol and ibuprofen there are 29 619 and 38 692 items indexed in WoS, respectively. The panel of drugs encompasses such diverse (from the mechanism’s standpoint) molecules as DNA and/or RNA synthesis inhibitors (5-FU and TDX),^29–32^ antifolate agent (MTX),^33^ tubulin-active antimitotic agent (PCTL),^34,35^ and free radical formation and DNA damage agent (DOXO).^36^ Here we assess by means of proteomics whether or not the similarity in the formally assigned mechanism of action leads to the similarity in the death/survival pathways.

The protein abundance changes in dying cells were analyzed to reveal the proteins intimately involved in drug action, including the probable drug targets (set S). The 'causal' set S represents the upstream part of the cellular death mechanics, affection of which ultimately leads to cellular demise. Using the GO terms for various biochemical processes, proteins were also grouped into sets B*_i_*, representing the proteins mediating the death cascades (*i* represented different GO terms). In parallel, the sets of *N* most abundant proteins (sets A*_N_*) were analyzed. These proteins, which are downstream of all death-related cascades, were taken to reflect the cellular morphological changes in the process of dying.

The two central questions of this study were: (i) which of the 'biochemical' sets B_i_ reflects best the changes in the 'causal' set S and (ii) how well is the 'morphological' set A reflects the behavior of set S? The hope was that, by answering the first question, we could evaluate the potential of proteomics for cell death modality discrimination. The second question would allow us to distinguish between the loose and tight models of cell death regulation, and simultaneously to verify the ability of 'quantitative morphology' (represented by the top proteome) to reflect the cell death mechanisms. In particular, the loose model predicts that at least some of the drugs would lead to practically indistinguishable morphological states of the cell, while the tight model suggests that all final cellular states will be well separated, and could potentially be traced back to the specific drug.

## Results

### Verification of the approach

**Figure 2:**
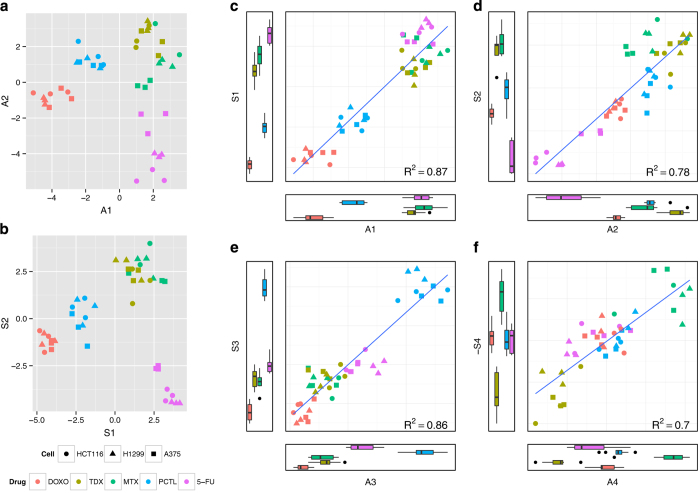
Comparison of two sets of proteins in terms of produced OPLS-DA models. (**a**) OPLS-DA model (first two dimensions are shown) separating different drug treatments for the A_100_ set encompassing 100 most abundant proteins. (**b**) The same for the S-set encompassing 100 most specific proteins, including all primary and secondary drug targets. (**c**–**f**) Correlations between the first four coordinates of the OPLS-DA models of the A_100_ set and S sets.

To verify that the S-set model for the three cell lines represents a good reference object, the protein 'loadings' of its OPLS-DA components were investigated (Figure 2). For the first (most significant) component that separates DOXO and PCTL on the one side from 5-FU, MTX and TDX on the other site (Figure 2a), the most typical DOXO representative was found to be ATP synthase ATP5A1, while the 5-FU champion was the RNA-binding motif protein RBM28. Furthermore, among the DOXO/PCTL-specific proteins, there is also a group of tubulins (PCTL targets), while TYMS (5-FU target) is found among the eight most 5-FU/TDX/MTX-specific proteins. The second OPLS-DA component separates the 5-FU and TDX/MTX treatments. Here, upregulation of eukaryotic translation initiation factor EIF4B and eukaryotic translation elongation factor EEF1B2, as well as downregulation of ribosomal protein RPL23A, were found to be most specific for 5-FU, while upregulation of DHFR (primary target for MTX and secondary target for TDX), TK1, CDK1 and PRIM1 were specific for TDX/MTX. Interestingly, TYMS was found in the middle group in this component, indicating that this protein is engaged in both 5-FU- and TDX/MTX-induced pathways. The third component (Figure 2e) separates PCTL from DOXO; upregulated tubulins and downregulated BPTF were specific for PCTL, while the DOXO-related proteins were CDK2 and SEC14L2. The fourth component (Figure 2f) differentiates TDX (the specific group of proteins includes CDK2, PRIM1, TK1 and TYMS) from MTX (SYNJ2, SEC14L2 and DHFR). These findings are in a broad agreement with existing knowledge on the targets and the mechanism of action of these drugs,^27,29–32,34,35,37^ which supports the validity of the S-set-based model.

As an example of the representativity estimate, the topologies of the OPLS-DA models for the sets S and A_100_ were compared (Figures 2c–f). The coordinates in all four orthogonal OPLS components correlate well with each other, with Pearson’s correlation ranging from 0.81 to 0.93. The representativity factor for this comparison was calculated to be 0.86 (0.7 after scaling).

### H1299 cell line

**Figure 3:**
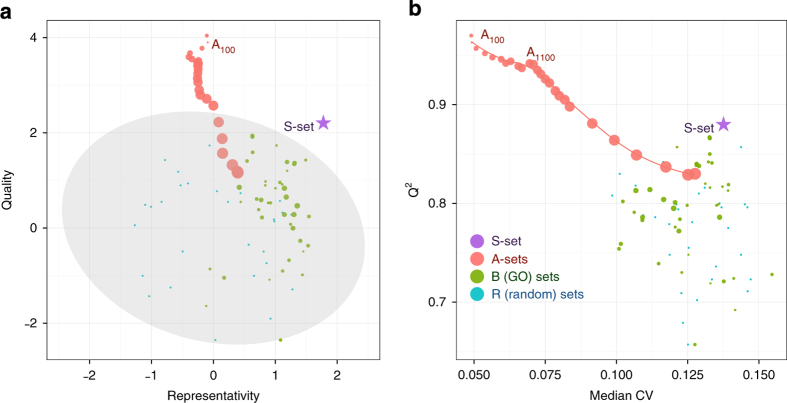
Quality and representativity of OPLS-DA models for different sets of proteins trained on proteomics data from H1299 cells treated with five drugs. (**a**) Normalized quality and representativity of different sets of proteins. The 95% confidence area for random sets is shown in gray. The circle sizes are proportional to the sizes of the protein sets. (**b**) Scatter plot of the OPLS-DA models’ *Q*^2^ values *versus* the median CV of protein abundances used in the model.

Figure 3a depicts the quality and representativity of various models based on the proteome changes in this cell line, while the model parameters are provided in Supplementary Table S2. All B-sets and A-sets for *N*>2400 fell into the shaded area representing random sets distribution (95% confidence). This fact indicates that even a randomly chosen proteomic set contains enough information to generate a good model separating the chosen treatments (mean *Q*^2^ for random sets was 0.77). This, in turn, means that the information on the drug-induced effects is largely spread over the whole proteome. Yet the A-sets up to a moderate size (*N*<2400) give better-quality models than any S-set.

Better performance of A-sets in terms of models’ quality (*Q*^2^) can at least partially be explained by that fact that more abundant proteins are usually more accurately measured. In the Figure 3b models’ *Q*^2^ are plotted against median CV of protein abundances in the data set. One can clearly see that the quality of the OPLS model decreases for the sets with higher median CVs. The good quality of A-sets in line with their relatively low representativity shows that morphological changes of cells under treatment are both drug-specific (good quality means that different treatments can be easily separated in the OPLS space) and cell-specific (upstream drug-specific perturbance of the proteome differs in terms of the OPLS topology from the downstream morphological changes). Thus, combining data on different cell lines could be away to separate drug-specific changes in morphology from cell-line-specific proteome responses.

### Three cell lines

**Figure 4:**
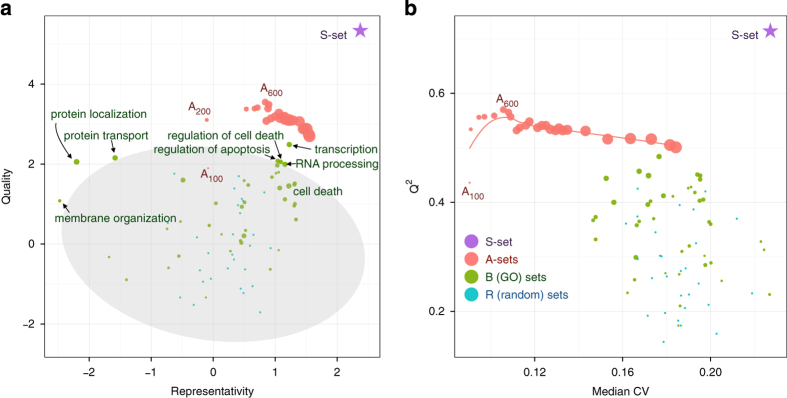
The same as in Figure 3 but for the combined data set on three cell lines (HCT116, A375 and H1299). (**a**) Normalized quality and representativity of different sets of proteins. The 95% confidence area for random sets is shown in gray. The circle sizes are proportional to the sizes of the protein sets. (**b**) Scatter plot of the OPLS-DA models’ *Q*^2^ values *versus* the median CV of protein abundances used in the model.

Combining the data for all three cell lines minimized the cell-line-specific variations and thus highlighted the drug-specific protein abundance changes. In Figure 4a, only very few B-sets were found to behave better than the random sets (see also Supplementary Table S3). Among the significant GO terms are the regulation of cell death, regulation of apoptosis, RNA processing and translation, while the protein transport and protein localization sets provided models with better quality but worse representativity. At the same time, all A-sets but one were found to be significant (better than random), while any A-set with *N*≥200 produced a better-quality model than any B-set.

As expected, the CVs for both single- and triple-cell data increased with *N*. However, unlike the single cell line data, the quality of OPLS models increased with *N* for A-sets from 100 to 600 proteins and then decayed slowly (Figure 4b). There can be two reasons for this. First, combining data from three cell lines makes models more tolerant (to some extent) to experimental variations. Second, more proteins are needed for a combined data set to build a good model to separate drug-specific morphological changes from cell-line-specific changes.

## Discussion

To answer the first central question of this study, the set B_12_ encompassing the GO term 'Transcription' reflected best the abundance changes of the set S, followed by B_7_ and B_8_ (death-related GO terms) as well as B_2_ (RNA processing). While the presence of the death-related terms among the significant B-sets was anticipated, the prominence of the transcription and RNA processing B-sets was a bit surprising, though it could be explained by the fact that both transcription and RNA processing proteins are found in the highly abundant part of the cellular proteome.^26^ A larger panel of drugs, and perhaps of the cell lines, would allow one to identify which biochemically active protein sets most faithfully reflect the drug action mechanism.

The most striking finding in this study is the exceptionally good performance of the 'morphological' A-sets for combined data, which superseded the B-sets both in terms of the model quality as well as the representation of the drug action mechanism. Taken at the face value, this finding means that the regulation pattern of the abundant, household proteins faithfully reflects that of the proteins interacting with the drug, in a broad agreement with the rigid cell death model predictions. However, the final verdict on the rigid *versus* loose death mechanism can only be given when the number of tested drugs will greatly exceed that of the possible death pathways, that is, at least a few dozen.

If further research validates the rigid regulation model, top proteome analysis may become a valuable tool in drug development. Given that the quantitative analysis of the top proteome (≥1000 proteins) can take as little as 10 min of instrumental time in a modern LC-MS/MS analysis, large-scale screening of drug action mechanisms may become possible.

## Materials and Methods

The data set for this study was taken from Chernobrovkin *et al*.^28^ In short, three cancer cell lines (melanoma A375, lung cancer H1299 and colon cancer HCT116) were treated in three biological replicates with five common anticancer agents, each one associated in literature with apoptosis: 5-FU,^29,30,38,39^ methotrexate (MTX),^39^ paclitaxel (PCTL),^34,35^ doxorubicin (DOXO)^37,40^ and tomudex (TDX).^30^ After 72 h treatment, most cells were dead (floating), with remaining cells dying but still attached. The attached dying cells were collected and lysed. Subsequent label-free proteomics analysis of cell lysates identified and reliably quantified 4168 proteins across all the treatments and cell lines.^28^ Most of the further analysis was done twice, first for the representative cell line (H1299) and then for the combined data on all three cell lines.

### Selection of set S

For each drug molecule, proteins were ranked according to the relation to the drug’s mechanism of action and the corresponding *P*-values were calculated for each protein and drug as in ref. 28. For each individual protein, the *P*-values were multiplied, and the proteins were sorted in ascending order of the product. Top 100 proteins with the lowest products were selected as set S (Supplementary Table S1). The set S encompasses all known protein targets for the drugs used, for example, TYMS for 5-FU and TDX, DHFR for MTX, ß-tubulins for PCTL.^28^

### Selection of sets B

Fourteen representative 'biochemical' sets (Table 1) were built using GO annotations (biological process) of groups encompassing 105–344 identified proteins. Protein identities in each B-set are given in Supplementary Table S1.

### Selection of sets A

All quantified proteins were sorted according to the reference abundance (geometric mean of integrated ion current of all unique peptides for all cell types and treatments)^41^ in descending order. Subsets encompassing *N*=100, 200, …, 4168 proteins were created and subjected to further analysis.

### Random sets

Sets of 100 proteins randomly chosen out of all quantified proteins were used to estimate the 'background level' of statistical significance of findings.

### GO annotation

DAVID tool^42^ was used to extract gene ontology annotation for each quantified protein.

### Data sets comparison

Multivariate approach was applied to reveal how different sets of proteins reflect cell behavior upon different treatments. The normalized and scaled protein abundances in each set were projected on latent structures using OPLS-DA^43^ method realized in SIMCA software (v. 13.5; Umetrics, San José, CA, USA). ‘UV scaling’ of variables was used leading to zero mean and unit variance for each protein across all samples. Classes were assigned to each sample according to drug treatment applied, namely 5-FU, DOXO, PCTL, MTX and TDX. Sevenfold cross-validation was used to determine *Q*^2^ of the model for each number of orthogonal components.^44^ The model based on the S-set was selected as a reference. All other models were compared with it in terms of the model quality (cross-validated *Q*^2^) and 'representativity' of the drug action mechanism. The latter parameter was estimated as topology similarity with the set S model, and determined as follows. Projection coordinates (scores) for each component of the model were correlated with the corresponding coordinates of the S-set model. The mean value of the correlation coefficient between the two sets of components was chosen to be the 'representativity' estimate. Finally, quality and representativity values were normalized so as to provide zero means and unit variances for the models built upon random sets each comprising 100 randomly chosen proteins.

## Figures and Tables

**Table 1:** Selected B-sets

*Set*	*GO term*	*Description*	*Number of proteins*
B_1_	GO:0006281	DNA repair	105
B_2_	GO:0006974	Response to DNA damage	138
B_3_	GO:0000278	Mitotic cell cycle	165
B_4_	GO:0016310	Phosphorylation	210
B_5_	GO:0006928	Cell motion	122
B_6_	GO:0007010	Cytoskeleton organization	148
B_7_	GO:0007049	Cell cycle	288
B_8_	GO:0008283	Cell proliferation	138
B_9_	GO:0010941	Regulation of cell death	251
B_10_	GO:0015031	Protein transport	344
B_11_	GO:0006412	Translation	231
B_12_	GO:0006350	Transcription	340
B_13_	GO:0008219	Cell death	224
B_14_	GO:0006915	Apoptosis	185
