# Supplementary material for: How well can morphology assess cell death modality? A proteomics study
Source: Cell Death Discov. 2016 Oct 3;2:16068–. doi: 10.1038/cddiscovery.2016.68 (PMC5045961; doi:10.1038/cddiscovery.2016.68)
Supplement: Supplementary Information [file cddiscovery201668-s1.doc]

**Title**

How well can morphology assess cell death modality? A proteomics study

**Authors/Affiliations**

Alexey Chernobrovkin and Roman A. Zubarev

Division of Physiological Chemistry I, Department of Medical Biochemistry and Biophysics, Karolinska Institutet, Scheelesväg 2, SE-17 177 Stockholm, Sweden

**Contact**:

Roman.Zubarev@ki.se

**SUPPLEMENTAL INFORMATION**

Table S1. Selected datasets (S, Bs and As).

Table S2. Properties of the OPLS-DA models trained on the proteomics data from the lung cancer H1299 cells.

Table S3. Properties of the OPLS-DA models trained on the proteomics data from melanoma A375, lung cancer H1299 and colon cancer HCT116 cells.
